# From the frontlines to the future: Anti-epidemic volunteer experience and career rewards for doctors

**DOI:** 10.1371/journal.pone.0328044

**Published:** 2025-07-22

**Authors:** Jing Qi, Dongyu Huang, Yiping Chen, Dingkai Huang, Zhijian Jiang

**Affiliations:** 1 Fujian Provincial Hospital, Fuzhou, Fujian, China; 2 School of Economics, Xiamen University, Xiamen, Fujian, China; Mediterranean University of Reggio Calabria: Universita degli Studi Mediterranea di Reggio Calabria, ITALY

## Abstract

Amid a series of sudden public health crises that have erupted since the beginning of the 21st century, healthcare professionals in China have consistently been recognized as frontline heroes. This study, based on unique data from a major hospital in China, focuses on doctors who volunteered during the COVID-19 outbreak in Wuhan, China. We examine the changes in remuneration and career trajectories of these volunteers upon their return to regular duties. Our findings reveal that these “heroes” received higher remuneration upon reintegration into their original workplaces, reflecting recognition for their sacrifices. Mechanism tests demonstrate that volunteer doctors are more likely to engage in roles requiring advanced technical expertise and receive priority in promotion processes upon returning. Moreover, we observe that male, highly educated, and experienced volunteer doctors receive greater remuneration, indicating the need for fairness in reward mechanisms. By elucidating the mechanisms and actual effects of career rewards for volunteer doctors, this study provides feasible suggestions and theoretical support for policymaking in future public health emergencies.

## 1 Introduction

In the contemporary era, the global landscape has been punctuated by recurrent public health emergencies, such as the emergence of SARS, H1N1 influenza, MERS, Ebola, and the pervasive COVID-19 pandemic. In China, the battle against these pathogens has prominently highlighted the pivotal role of healthcare professionals. Amidst such crises, healthcare personnel proactively make significant personal sacrifices, deploying their expertise at the vanguard of disease mitigation efforts. They endure formidable workloads and prolonged isolation from their families and friends, which imposes immense physical and psychological strain [1]. Moreover, they directly confront the virus in close quarters, bearing the brunt of infection risk, while also managing the emotions of patients and the expectations of their families [2]. Extensive empirical evidence has illuminated the adverse impact of the highly transmissible COVID-19 on the mental health of healthcare workers, with enduring effects post-outbreak [1, 2], manifesting as depression, anxiety, and sleep disturbances [3, 4].

What motivates doctors to take significant risks during public health crises? Understanding this phenomenon requires delving into the realms of psychology and behavioral economics. Psychology suggests that this behavior is closely related to the concept of altruism, where individuals engage in volunteer activities to fulfill their desire to contribute to the common good [5], thereby achieving self-worth and satisfying their spiritual needs. Behavioral economics offers another perspective, proposing that during crises, the altruistic drive of doctors often surpasses economic incentives, leading them to prioritize patients’ health over their personal financial gains [6].

Evidently, healthcare professionals stand as humanity’s bulwark in public health exigencies, likened to “superheroes” amidst crises. In China, healthcare professionals have been widely celebrated as “heroes” for their contributions during public health crises, particularly the COVID-19 pandemic. A salient inquiry emerges: post-crisis, do these erstwhile “superheroes” garner societal or organizational “rewards” despite their initial non-pecuniary motivations? If affirmative, what modalities do these “rewards” assume? Constrained by the scarcity of accessible data, the field of health economics currently lacks sufficient empirical research evidence on this matter. This gap motivates our study to examine how Chinese healthcare professionals’ contributions during the COVID-19 pandemic translated into tangible rewards from their organizations and society. Existing literature underscores the significant influence of public health emergencies on healthcare workers’ psychological well-being and career trajectories [2, 7–9]. However, limited research explores post-crisis career development and compensation outcomes for healthcare workers.

The sudden outbreak of the COVID-19 pandemic in 2020 posed a grave threat to human health, and impeded global socio-economic progress. In January 2020, Wuhan was suddenly struck by the COVID-19 pandemic. Tasked with addressing this emergent and hitherto unparalleled pathogen, the local healthcare system experienced acute resource constraints, endeavoring to marshal a robust and effective response. In this critical situation, numerous healthcare volunteers from across China converged on Wuhan, braving personal peril to deliver timely treatment and meticulous care to patients in need. We obtained individual-level data from a large hospital (with a staff of 1,090 doctors) in China, including 44 doctors who volunteered to fight the epidemic in Wuhan. This study seeks to probe the career “rewards” (i.e., increased base salaries and performance-based bonuses) accorded to these altruists upon returning to their workplaces.

Our analysis discerns that the intrepid Wuhan volunteers were subsequently accorded augmented remuneration (both in base salaries and performance-based bonuses) by their employers, affirming that these “superheroes” have not faded into oblivion. Rigorous robustness checks substantiate our findings. Upon returning to their workplaces, the volunteer doctors were poised for roles demanding elevated professional acumen (e.g., surgical treatment) and were prioritized for career advancement, reflecting their valor and dedication have been recognized. Furthermore, disparities in remuneration favoring male, experienced, and highly educated volunteer doctors, suggesting the imperative for a recalibrating of reward mechanisms to ensure equitable distribution.

Compared to the existing research, our study makes the following marginal contributions: First, by utilizing unique individual-level data, we delve into whether doctors’ personal dedication during the public health crises gains the rewards. We confirm a positive relationship between doctors’ volunteer engagements and subsequent professional remuneration, thereby extending the findings of existing studies. Second, we underscore the gender disparities in career progression. Even amidst the global emergency of combating a pandemic, the rewards women received are still less than those of their male counterparts under equivalent circumstances. It indicates that further efforts are necessary to ensure gender equality, safeguarding women’s rights in career trajectories. Third, in light of the increasing risk of pandemic outbreaks, it contributes a nuanced discourse on reward measures for healthcare professionals in the face of escalating pandemic risks.

## 2 Literature review

Health economics traditionally casts doctors as agents of self-interest, inclined to optimize personal financial returns in service delivery. In ordinary times, doctors’ motivations are often driven by economic rewards and career advancement opportunities. For instance, the Fee-For-Service (FFS) paradigm incentivizes additional care provision to augment doctor income, often culminating in service overutilization [10, 11]. In contrast, under a Capitation (CAP) payment system, doctors receive a fixed payment, which typically curtail service supply [12]. A study demonstrated that Medicare’s elevated doctor reimbursement spurred diagnostic imaging (such as MRIs) utilization and reduced cardiac mortality, highlighting the pivotal role of doctor incentives in healthcare delivery and patient welfare [13]. Additionally, factors such as housing security and long-term employment contracts also stand out as particularly significant [14, 15].

However, the altruistic behavior exhibited by doctors during crises is particularly noteworthy. Understanding doctors’ motivations during such periods can be explored from multiple perspectives, including psychology and behavioral economics. Psychological theories often emphasize intrinsic motivations, such as the desire to help others, which are closely related to the concept of altruism. Altruistic behavior is typically associated with significant psychological benefits [16]. Individuals frequently engage in volunteer activities to fulfill their desire to contribute to the common good [5]. During the COVID-19 pandemic, doctors demonstrated remarkable dedication, even under extreme working conditions [17]. Behavioral economics provides another lens, suggesting that during crises, the drive of altruism often outweighs extrinsic economic incentives. Research shows that doctors are more influenced by intrinsic altruism in such times, prioritizing patients’ health over their own financial gains [6]. This phenomenon has been observed globally, particularly in the hardest-hit regions where doctors often voluntarily take on more work and even risk their own health to care for patients.

The literature seldom spotlights the altruistic doctor subset volunteering in epidemic hotspots and conflict zones, despite their exposure to mortality risks and attendant mental health challenges. Post-COVID-19, studies have delineated the pandemic’s toll on healthcare workers’ psychological state, exacerbating anxiety and attrition propensities as patient loads swell [3, 7, 9]. It invites contemplation of the considerable strain and profound personal sacrifices that volunteer doctors are compelled to endure at the onset of an epidemic, when confronting an unfamiliar and virulent disease. For example, a study by [18] focusing on doctors in Wuhan during the initial COVID-19 outbreak found that up to half of the healthcare workers experienced symptoms of anxiety and depression. While profit-motivated doctors may calibrate service provision to expected returns, volunteers are driven by non-remunerative motives. Following the resolution of a crisis, it is uncertain whether the selflessness exhibited by these medical professionals will be eclipsed by time or commemorated with lasting “rewards” from their organizations or the broader society. This area remains underexplored in academic literature, largely attributable to constraints on data accessibility.

In the context of sudden public health emergencies, existing research indicates that women are more inclined to engage in jobs with significant social impact [19]. Female healthcare workers often undertake high-contact nursing roles during pandemics, facing higher infection risks, and as a result, they may receive increased remuneration [20, 21]. Conversely, literature also supports that male healthcare workers, due to societal expectations of gender roles, are more frequently assigned to critical positions requiring intense labor and rapid decision-making, or take on more pandemic-related tasks. This allocation aligns with the stereotype that men are “more competent and authoritative” in handling critical matters [22], with such roles often linked to higher performance evaluations and economic incentives [23]. Therefore, even under equivalent circumstances, male volunteer doctors are more likely to be assigned to higher-skilled jobs, resulting in specific salary benefits. Furthermore, male doctors may have broader career advancement opportunities after participating in the Wuhan anti-epidemic rescue, especially in ascending to managerial or leadership positions. When performance evaluation criteria are ambiguous, gender biases in evaluation systems may unconsciously elevate male healthcare workers’ performance scores, contributing to higher salary levels [24, 25].

From the perspective of human resource management, doctors with higher educational qualifications generally possess a stronger theoretical foundation and enhanced learning capabilities. This enables them to provide superior medical services during critical situations such as pandemics [26]. Additionally, their research background and rapid learning capabilities provide an edge in developing and implementing new treatment protocols, a crucial advantage during pandemics [27]. Therefore, highly educated doctors are more likely to be assigned to roles that demand advanced professional skills, resulting in greater salary returns following their volunteer efforts. Hospitals’ internal promotion mechanisms and pay structures may also positively impact salary growth for highly educated doctors [28].

In the medical field, doctors with more years of service play a crucial role in crisis management due to their clinical experience and deep expertise. Studies have shown that in emergency situations, more seasoned doctors exhibit faster decision-making speeds and higher accuracy, which helps improve efficiency and reduce patient waiting times [29]. Their long-term clinical practice allows them to handle emergency situations efficiently and meet complex challenges effectively. Consequently, seniority is often an important consideration in job assignments and promotions. Additionally, seasoned doctors enjoy higher professional prestige within the medical community, with their contributions more likely recognized by hospital evaluation systems, often translating to higher performance scores and corresponding salary increases [30].

## 3 Hypothesis development

Since the 21st century, the world has witnessed multiple large-scale pandemics. When societies are threatened by diseases, medical volunteer teams are often at the vanguard of the response. After successfully managing these health crises, these volunteers return to their regular positions, continuing their dedication to public health. We propose that these valiant doctor volunteers, though driven by altruistic motives rather than utilitarian ones, receive career rewards for their actions, often beyond their initial expectations.

First, doctor volunteers who confront high-risk infectious diseases directly gain invaluable professional experience that significantly enhances their skills, thereby increasing their career prospects. During the COVID-19 pandemic, for example, healthcare workers faced heightened infection risks and psychological stresses due to necessary measures to curb virus transmission and care for the infected [31]. Through prolonged exposure to high-intensity work and heightened infection risks, these volunteer doctors gained deeper insights into the pathological mechanisms, treatment methods, and prognoses of related diseases, while rapidly developing skills to manage diverse medical emergencies [32]. Furthermore, in hard-hit areas with scarce medical resources, they endured long hours of intense work under high-risk conditions [4]. Such demanding conditions enhanced their expertise in emergency management and bolstered their resilience under pressure. These enhancements not only boost clinical competencies but also increase their value within their organizations. In medical care, outpatient services typically involve routine health checks and chronic disease management, often addressing less severe conditions. In contrast, inpatient care requires overnight hospital stays for complex surgeries, emergency treatments, or continuous monitoring, demanding greater clinical expertise and experience [33]. Post-pandemic, it is plausible that doctors who volunteered in Wuhan would take on roles requiring higher technical skills, resulting in a decrease in outpatient performance income and an increase in inpatient performance income. Given the specialized nature of these tasks, such shifts could translate to greater performance-based rewards, ultimately elevating the overall remuneration of these doctors.

Second, within the healthcare system, doctors are differentiated by their professional titles, and volunteer experiences provide distinct advantages for career progression. Career promotions recognize outstanding contributions in current roles [34] and active involvement in community service [35–37]. High-risk, high-pressure roles, like those in anti-epidemic rescue, are particularly valued for demonstrating resilience, leadership, and professional competence. Consequently, volunteer doctors tend to advance more rapidly than peers without such experiences. Additionally, salary increases for doctors involved in Wuhan’s anti-epidemic efforts reflect formal recognition and rewards for their frontline altruism. Therefore, we anticipate that doctors who participated in the Wuhan anti-epidemic rescue are likely to experience enhanced career opportunities and increased salary levels post-pandemic.

By integrating these two pathways—skills enhancement and career advancement—we hypothesize that doctors with anti-epidemic volunteer experience will achieve economic gains through elevated salaries, encompassing both base pay and performance-based bonuses.

**H1:** Doctors with anti-epidemic volunteer experience are more likely to receive an increase in their remuneration.

## 4 Methods and research design

### 4.1 Data and sample

In January 2020, as COVID-19 emerged, doctors from across China formed volunteer teams and converged on Wuhan to combat this lethal virus. After effectively controlling the outbreak, these volunteer doctors returned to their original workplaces. Our study explores the existence of career “rewards” for these volunteers. We obtained the anonymized personal data of doctors from the financial database of a major hospital in China, covering a period from 2017 to 2023. The dataset encompasses a comprehensive total of 1,090 doctors, including general practitioners, specialists, and residents. The selection of volunteers for the Wuhan anti-epidemic response was conducted during an emergency, where the hospital issued a public notice inviting all doctors to volunteer simultaneously. Once the number of volunteers required was determined, the hospital promptly disseminated information through various departments, emphasizing the urgency of the situation and following a first-come, first-served principle. This ensured that all doctors received clear and timely information about the support mission. Afterwards, the registration process was initiated and applications were collected to form the volunteer team based on availability and suitability. We obtained authorization to access the dataset on March 29, 2024, and all personally identifiable information had been anonymized prior to the access and analysis. Due to the respiratory nature of COVID-19, our sample was limited to departments related to this specialty. We excluded observations with missing values for variables and all continuous variables are winsorized at the upper and lower 1 percentiles of the sample distribution. We obtain a preliminary dataset of 3,619 doctor-year observations, including 560 doctors. Robustness tests were conducted across samples from all departments to confirm the generalizability of our findings.

### 4.2 Statistical analysis

#### 4.2.1 Baseline model.

To verify the impact of volunteer participation in Wuhan’s COVID-19 response on the future career returns of doctors, we employed a difference-in-differences (DID) model, based on prior literature:

$${Earnings}_{i,t}=\alpha +\beta_{0}treat\times {post}_{i,t}+\beta_{k}\sum_{k=1}^{K}{Control}_{i,t}+\gamma_{t}+\theta_{i}+\varepsilon_{i,t}$$ (1)

*Earnings*_*i*,*t*_ represents the doctors’ remuneration levels, which are measured as follows: (1) *salary*, the logarithm of base salary, which is determined by the doctor’s years of service and professional title; (2) *bonus*, the logarithm of performance-based bonuses, which is related to the doctor’s workload as well as the complexity and specialization of their work; (3) salary_bonus, the logarithm of the combined base salary and performance-based bonuses. The interaction term treat×post is our key explanatory variable, treat divides the doctor samples based on whether they participated in the Wuhan anti-epidemic rescue, with the treated group comprising the volunteers and the control group consisting of their non-volunteering colleagues from the same department during the same period [38]. *Post* is assigned a value of 1 for samples from 2020 onward (post-outbreak) and 0 otherwise. It is worth noting that the model includes year fixed effects $\left(\gamma_{t}\right)$ and individual fixed effects $\left(\theta_{i}\right)$, thus there is no need to separately include the *treat* and *post* variables, as they will be absorbed by the fixed effects and ensuring that time-invariant characteristics such as gender, education, and other personal traits that do not change over time are absorbed by the fixed effects. More detailed descriptions of all variables are summarized in Table 1, and the standard errors are clustered at the department level.

**Table 1:** Variables’ definition.

Varibles	Definition
*salary*	Logarithm of base salary
*bonus*	Logarithm of base salary and performance-based bonuses
*salary_bonus*	Logarithm of combined base salary and performance-based bonuses
*treat*	Indicator for doctors participating in the Wuhan anti-epidemic rescue: 1 if participated, 0 otherwise
*post*	Indicator for years post-2020 pandemic: 1 for 2020 and beyond, 0 otherwise
*workload*	Average annual workload, logarithm of annual outpatient visits
*seniority*	Logarithm of seniority
*age*	Logarithm of age
*outpatient*	Logarithm of outpatient performance bonuses
*inpatient*	Logarithm of inpatient performance bonuses
*dtitle*	Indicator for professional title increased after 2020: 1 if increased, 0 otherwise
*dadministration*	Indicator for administrative level increased after 2020: 1 if increased, 0 otherwise
*education*	Educational level: 1 for undergraduate and below, 2 for Master’s degree, 3 for Doctoral degree
*gender*	Gender: 1 for female, 0 for male

#### 4.2.2 Robustness analysis.

Initially, the parallel trends assumption, which is a prerequisite for conducting DID model, allows for an assessment of trend changes between the treated and control groups before and after policy implementation. To test whether there were pre-existing remuneration differences between the treatment and control group companies before the volunteer participation in Wuhan’s COVID-19, we conducted parallel trend assumption tests for multi-period difference-in-differences. The regression model is designed as follows:

$${Earnings}_{i,t}=\alpha +\sum_{n=-3;n\neq -1}^{n=3}\mu_{n}{postyear}_{i,n}+\beta_{k}\sum_{k=1}^{K}{\mathrm{Control}}_{i,t}+\gamma_{t}+\theta_{i}+\varepsilon_{i,t}$$ (2)

The model setup is essentially the same as Model (1), with the only difference being the specification of the interaction term. *postyear*_*i*,*n*_ represents the nth year of supporting Wuhan, with the year before the pandemic (i.e., 2019, n=−1 ) as the base period. $\mu_{n}$ is the coefficient of the explanatory variable of interest in the parallel trend test. When *n*<–1, if $\mu_{n}$ is not significantly different from 0 , it indicates that there is no significant difference in salaries between the treatment and control groups before the volunteer activities, satisfying the parallel trend assumption.

In addition, we employed the Propensity Score Matching-Difference in Differences (PSM-DID) method to better estimate the treatment effect and control for potential endogeneity issues arising from “selection bias.” Propensity Score Matching is a commonly used statistical method in observational studies. It first matches the treated group with a control group that is as similar as possible based on propensity scores, and then uses the difference-in-differences approach to obtain the average treatment effect of the policy, thus making the results more reliable. Due to space limitations, we have placed the results of this test in the supporting information.

Furthermore, we conducted a placebo test by randomly reassigning the group of doctors who participated in the Wuhan anti-epidemic response and the timing of their involvement. We then re-ran the regression model 1,000 times using this randomized data. Given that the assignment of doctors to the treated group and the timing of their participation were randomly determined, the placebo test should not reveal any significant impact on the doctors’ salary and bonus, indicating that the regression coefficients should be close to zero. Due to space limitations, we have placed the results of this test in the supporting information.

After that, we attempt different model structures or estimation methods, such as altering the fixed effects of the model or employing various clustered standard errors, to prevent result biases that may arise from incorrect model specifications.

#### 4.2.3 Subsample analysis.

To account for potential biases stemming from prior experiences with public health crises, we conducted a supplementary analysis to exclude the impact of SARS on individuals’ perceptions of volunteering and rewards. Specifically, we restricted the sample to doctors born after 1985, who were not yet university students when SARS broke out in 2002. This group was less likely to have formed associations between volunteering in health crises and receiving rewards, as compared to those who were medical students or already working during the SARS period. While some recognition of healthcare workers’ contributions occurred during previous epidemics, such as SARS, the explicit linkage between volunteering and rewards became more pronounced during the COVID-19 pandemic. This shift can be attributed to the unprecedented scale of the COVID-19 pandemic, heightened societal expectations, and the formalization of reward mechanisms by organizations.

Additionally, we conducted a subsample analysis to test the robustness of our results. We included both those who were selected for the treatment and those who volunteered but were not selected (the runner-up group).

#### 4.2.4 Mechanism analysis.

Mechanism analysis is an important means of identifying causal relationships and helps to strengthen the argument for the causal relationship from voluntary activities to rewards. Following Dell (2010) [39], we sequentially replace the dependent variable with outpatient income (*outpatient*), inpatient income (*inpatient*), professional title promotion (*dtitle*), and administrative level increase (*dadministration*) to test the channels of influence. The detailed results of this analysis are presented in Sect 5.6.

#### 4.2.5 Heterogeneity analysis.

Given that the impact of voluntary activities on rewards may vary across different individuals, we conducted a heterogeneity analysis to explore these effects. Specifically, we divided the sample into subsamples based on gender (*male*,*female*), educational level (*Ph*.*D*.,*non*–*Ph*.*D*.), and seniority (based on the median years of service), and conducted tests to identify differences between the groups. This heterogeneity analysis helps provide a deeper understanding of how different individual characteristics interact with the treatment and influence the variation in treatment effects. The results are presented in Sect 5.7.

## 5 Results

### 5.1 Descriptive statistics

Table 2 presents the descriptive statistics for the main variables. The mean of the logarithm of base salary (*salary*) is 10.591, while the mean for the logarithm of performance-based bonuses (*bonus*) is 11.946. This indicates that performance-based bonuses constitute a higher proportion of the total remuneration structure for doctors. The standard deviation for the logarithm of performance-based bonuses is 0.57, with minimum and maximum values of 8.739 and 13.124, respectively, suggesting that performance-based bonuses more significantly reflect variations in doctors’ remuneration. The mean value of *treat* is 0.083, indicating that approximately 8.3% of the doctors in the sample participated in frontline anti-epidemic rescue.

**Table 2:** Descriptive statistics.

Variable	N	Mean	SD	Min	p50	Max
*salary*	3619	10.591	0.387	8.811	10.483	11.474
*bonus*	3619	11.946	0.570	8.739	11.995	13.124
*salary_bonus*	3619	12.197	0.464	10.158	12.216	13.277
*treat*	3619	0.083	0.276	0.000	0.000	1.000
*post*	3619	0.600	0.490	0.000	1.000	1.000
*workload*	3619	5.825	2.503	0.000	6.538	9.385
*seniority*	3619	2.232	0.872	0.000	2.197	3.638
*age*	3619	3.633	0.212	3.219	3.584	4.078
*outpatient*	3619	6.873	2.803	0.000	7.677	10.919
*inpatient*	3619	11.914	0.567	9.421	11.964	13.149
*dtitle*	3105	0.143	0.350	0.000	0.000	1.000
*dadministration*	3105	0.023	0.151	0.000	0.000	1.000
*education*	3619	1.805	0.696	1.000	2.000	3.000
*gender*	3619	0.443	0.497	0.000	0.000	1.000

### 5.2 Remuneration trend analysis

If the decision to the Wuhan anti-epidemic rescue was not a voluntary choice by the doctors, but rather made under non-random circumstances, particularly favoring those doctors who were likely to experience rapid salary growth regardless of their participation in the anti-epidemic rescue, then the “Difference in Differences” estimation might produce a biased estimate of the causal effect. For example, this scenario could occur if hospitals strategically selected target doctors who either had stronger professional capabilities or were expected to have rapid future growth in salary and bonus to participate in the Wuhan anti-epidemic rescue. Although we cannot completely rule out this anticipatory selection, our study attempts to test this by comparing the salary differences trends between the treated group and the control group several years before and after their participation in the Wuhan anti-epidemic rescue.

**Fig 1:**
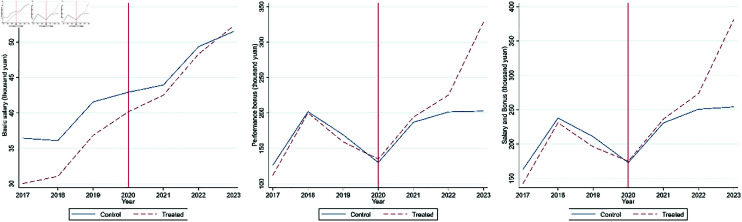
Remuneration trends of treated group and control group.

Fig 1 reports the average salary indicators for the treated and control groups from 2017 to 2023 and illustrates them with line graphs. From panels (2) and (3) of Fig 1, we observe that prior to the impact, the trends in performance-based bonuses and total remuneration for the treated and control groups were almost identical, with no significant differences. The situation in panel (1) of Fig 1 could be driven by the fact that frontline positions were more likely filled by general practitioners rather than leading specialists, and general practitioners generally have lower base salary levels. After 2020, it is evident that the salary and performance growth rates for the treated group were significantly higher than those for the control group. This test preliminarily confirms the impact of participation in the Wuhan anti-epidemic rescue on doctors’ salary and performance levels.

### 5.3 Baseline results

Table 3 presents the baseline regression results. The coefficients for doctors’ remuneration levels, measured in three different ways, are all positive and statistically significant at the 1% level after controlling for individual fixed effects, year fixed effects, and other relevant variables. This indicates a significant positive impact of voluntary participation in the Wuhan anti-epidemic efforts on doctors’ career returns. Specifically, doctors who with anti-epidemic volunteer experience received significantly higher salaries compared to their colleagues who did not engage in frontline work, thereby validating our hypothesis that volunteer doctors received career “rewards” from societal or organizational sources.

**Table 3:** Baseline regressions.

	(1)	(2)	(3)
	*salary*	*bonus*	*salary_bonus*
*treat×post*	0.0818^***^	0.1879^***^	0.1683^***^
	(0.0201)	(0.0569)	(0.0382)
*workload*	0.0041*	0.0534^***^	0.0408^***^
	(0.0020)	(0.0118)	(0.0081)
*seniority*	-0.0978^***^	0.4594^***^	0.3042^***^
	(0.0140)	(0.0558)	(0.0347)
*age*	1.7176^***^	2.0490^**^	1.5568^***^
	(0.4608)	(0.8705)	(0.5034)
*_cons*	4.5421^**^	3.1567	5.6165^***^
	(1.6546)	(3.1393)	(1.8172)
Individual Fe	Yes	Yes	Yes
Year Fe	Yes	Yes	Yes
N	3619	3619	3619
Adjusted ${\text{R}}^{2}$	0.9625	0.6983	0.7980

Note: (1) The significance levels of ***, ** and * are 1%, 5% and 10% respectively; (2) The standard error values are in brackets, and the model standard error is adjusted by clustering at the individual level. The following tables are the same; (3) Gender has been absorbed by the individual fixed effects, so it is not reported in the baseline regression.

### 5.4 Robustness tests

#### 5.4.1 Test of the parallel trend assumption.

**Fig 2:**
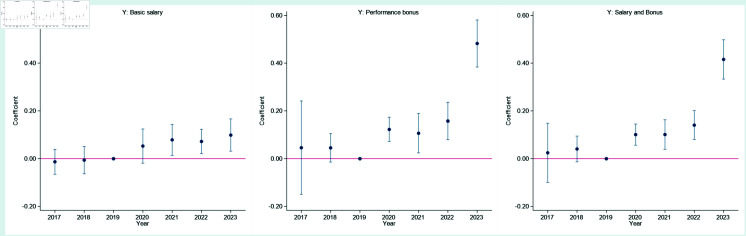
Parallel trend test.

The prerequisite for causal identification using a Difference-in-Differences model is the satisfaction of the parallel trend assumption. Fig 2 shows that before the outbreak of COVID-19, there were no significant differences in basic wages and performance-based bonuses between doctors who volunteered in Wuhan and those who did not (consistent with the parallel trend assumption). However, after the outbreak, significant differences emerged in both basic salaries and performance-based bonuses, with a larger change in performance-based bonuses. This suggests that the “rewards” were largely due to changes in skills, and that these rewards became more pronounced in the medium to long term.

#### 5.4.2 Adding fixed effects.

Considering that the salary levels of doctors might be influenced by the department’s workload over different periods, we introduced fixed effects for the interaction between department and year to our existing model with individual and annual fixed effects. This addition helps control for time-varying unobservable factors at the department level, such as changes in departmental dynamics. The specific results are reported in columns (1) to (3) of Table 4. After controlling for these high-dimensional fixed effects, the coefficient of the interaction term remains positive and statistically significant, further supporting the conclusions drawn earlier.

**Table 4:** Robustness test of adding fixed effects.

	(1)	(2)	(3)
	*salary*	*bonus*	*salary_bonus*
*treat×post*	0.0750^***^	0.1667^***^	0.1512^***^
	(0.0136)	(0.0558)	(0.0359)
*workload*	0.0030	0.0590^***^	0.0451^***^
	(0.0020)	(0.0107)	(0.0069)
*seniority*	-0.0908^***^	0.4389^***^	0.2909^***^
	(0.0146)	(0.0529)	(0.0309)
*age*	1.6264^***^	1.5288	1.1642^*^
	(0.4994)	(1.1237)	(0.6509)
*_cons*	4.8647^**^	5.0606	7.0484^***^
	(1.7929)	(4.0533)	(2.3518)
Individual Fe	Yes	Yes	Yes
Department-Year Fe	Yes	Yes	Yes
N	3619	3619	3619
Adjusted ${\text{R}}^{2}$	0.9642	0.7207	0.8120

#### 5.4.3 Changing clustering methods.

Previously, our estimates were based on robust standard errors clustered at the individual level, which helps mitigate possible correlations within cross-sectional or time-series dimensions. However, to more precisely capture the true variability of the estimated coefficients, we adopted robust standard errors clustered by both individual and year. This dual clustering more effectively addresses two-dimensional correlations. The specific results are reported in columns (1) to (3) of Table 5. The coefficients of the interaction term remain significantly positive, supporting the conclusions drawn earlier.

**Table 5:** Robustness test of changing clustering methods.

	(1)	(2)	(3)
	*salary*	*bonus*	*salary_bonus*
*treat×post*	0.0818^***^	0.1879^*^	0.1683^**^
	(0.0150)	(0.0972)	(0.0791)
*workload*	0.0041^*^	0.0534^***^	0.0408^***^
	(0.0022)	(0.0108)	(0.0070)
*seniority*	-0.0978^***^	0.4594^***^	0.3042^***^
	(0.0127)	(0.1103)	(0.0561)
*age*	1.7176^***^	2.0490	1.5568
	(0.4263)	(2.4678)	(1.7527)
*_cons*	4.5421^***^	3.1567	5.6165
	(1.5292)	(9.2231)	(6.4901)
Individual Fe	Yes	Yes	Yes
Year Fe	Yes	Yes	Yes
N	3619	3619	3619
Adjusted ${\text{R}}^{2}$	0.9625	0.6984	0.7981

#### 5.4.4 Alternative samples.

To further address concerns about sample selection, we expanded our analysis to include samples from all departments, revisiting the impact of voluntary participation in the Wuhan anti-epidemic rescue on the salary changes of individual doctors. The specific results are shown in columns (1) to (3) of Table 6. The coefficient of the interaction term remains significantly positive at the 1% level, further supporting the conclusions previously drawn.

**Table 6:** Robustness test of alternative samples.

	(1)	(2)	(3)
	*salary*	*bonus*	*salary_bonus*
*treat×post*	0.0789^***^	0.2283^***^	0.1981^***^
	(0.0215)	(0.0450)	(0.0315)
*workload*	0.0056^***^	0.0626^***^	0.0487^***^
	(0.0015)	(0.0044)	(0.0030)
*seniority*	-0.0791^***^	0.4544^***^	0.2975^***^
	(0.0105)	(0.0389)	(0.0249)
*age*	1.3487^***^	1.2644^**^	0.8834^**^
	(0.1991)	(0.6093)	(0.3862)
*_cons*	5.8414^***^	5.9219^***^	8.0041^***^
	(0.7071)	(2.1558)	(1.3664)
Individual Fe	Yes	Yes	Yes
Year Fe	Yes	Yes	Yes
N	6880	6880	6880
Adjusted ${\text{R}}^{2}$	0.9690	0.7026	0.7968

### 5.5 Excluding alternative explanations

First, we selected a subsample of doctors born after 1985 to exclude the pre-existing association between voluntary activities and rewards formed due to public health events like SARS, as these doctors had not yet entered university or started working during the SARS period. Our results, presented in Table 7, indicate that even after excluding the influence of SARS-related experiences, volunteer participation during the COVID-19 pandemic led to significant increases in salary (0.0435), bonus (0.2174), and the combined measure of salary_bonus (0.1855). The results indicate that while these workers were compensated for their contributions, their decision to volunteer was driven primarily by a sense of duty and the desire to help, rather than by extrinsic rewards.

**Table 7:** Excluding the potential cognition of volunteering and rewards.

	(1)	(2)	(3)
	*salary*	*bonus*	*salary_bonus*
*treat×post*	0.0435^***^	0.2174^**^	0.1855^***^
	(0.0066)	(0.0885)	(0.0594)
*workload*	0.0004	0.0614^***^	0.0456^***^
	(0.0009)	(0.0135)	(0.0086)
*seniority*	-0.0222^***^	0.3723^***^	0.2780^***^
	(0.0061)	(0.1128)	(0.0616)
*age*	-2.2500^***^	5.0285	3.8036
	(0.5637)	(7.1070)	(4.3871)
*_cons*	18.0866^***^	-6.5167	-1.8216
	(1.9429)	(24.4109)	(15.0690)
Individual Fe	Yes	Yes	Yes
Year Fe	Yes	Yes	Yes
N	1546	1546	1546
Adjusted ${\text{R}}^{2}$	0.9446	0.6299	0.6966

Second, we compared those who were selected with those who volunteered but were not selected (e.g., runners-up), providing additional insights into our results. As shown in Table 8, the results for the runner-up group also remain significant, with positive effects on salary (0.0968), bonus (0.2383), and salary_bonus (0.1955). This suggests that the rewards extended beyond the directly treated individuals to those who expressed willingness to volunteer, further supporting the robustness of our findings.

**Table 8:** Runner-ups comparison.

	(1)	(2)	(3)
	*salary*	*bonus*	*salary_bonus*
*treat×post*	0.0968^***^	0.2383^**^	0.1955^**^
	(0.0289)	(0.1048)	(0.0699)
*workload*	0.0121	0.0324^*^	0.0283^*^
	(0.0074)	(0.0179)	(0.0139)
*seniority*	-0.0897^**^	0.4012^**^	0.2169^**^
	(0.0349)	(0.1620)	(0.0799)
*age*	1.0141	2.7889	2.6861
	(0.8286)	(3.3248)	(1.7830)
*_cons*	7.0070^**^	0.7173	1.7769
	(2.9506)	(11.7831)	(6.3520)
Individual Fe	Yes	Yes	Yes
Year Fe	Yes	Yes	Yes
N	497	497	497
Adjusted ${\text{R}}^{2}$	0.9194	0.6807	0.7807

### 5.6 Mechanism test

Our theoretical analysis indicates that the remuneration of doctors who volunteered in Wuhan experienced a substantial increase post-2020, likely due to enhancements in their professional skills and career advancements.

#### 5.6.1 Professional skill enhancement.

To test the professional skill enhancement mechanism, we used two variables, *outpatient* and *inpatient*, representing the logarithm of outpatient and inpatient performance, respectively, to replace the dependent variable in the basic regression model, reflecting the changes in the income structure of doctors. The results, as shown in columns (1) and (2) of Table 9, indicate that the coefficient for treat×post in outpatient income is significantly negative, while it is significantly positive for inpatient income. This suggests a decrease in outpatient income but an increase in the inpatient portion, indicating that doctors involved in the Wuhan anti-epidemic efforts likely shifted towards more inpatient treatments. This shift reflects a reallocation of responsibilities aligned with their enhanced professional skills and resilience, leading to increased rewards. Therefore, the experience gained from voluntary participation in the Wuhan anti-epidemic significantly enhances doctors’ salary performance by improving their professional competencies [40].

**Table 9:** Mechanism tests.

	(1)	(2)	(3)	(4)
	*outpatient*	*inpatient*	*dtitle*	*dadministration*
*treat×post*	-0.0065	0.1877^***^	0.0466^*^	0.0301^***^
	(0.0365)	(0.0545)	(0.0261)	(0.0078)
*workload*	1.0931^***^	0.0434^***^	0.0036	-0.0002
	(0.0187)	(0.0095)	(0.0073)	(0.0006)
*seniority*	0.0008	0.4741^***^	0.0922	-0.0366^***^
	(0.0482)	(0.0511)	(0.0620)	(0.0071)
*age*	0.4117	1.8564^**^	3.4887^***^	2.2731^***^
	(0.3988)	(0.8392)	(0.7997)	(0.4277)
*_cons*	-0.9913	3.8501	-12.8074^***^	-8.1777^***^
	(1.4062)	(3.0216)	(2.8355)	(1.5523)
Individual Fe	Yes	Yes	Yes	Yes
Year Fe	Yes	Yes	Yes	Yes
N	3619	3619	3105	3105
Adjusted ${\text{R}}^{2}$	0.9906	0.6904	-0.0477	0.1380

#### 5.6.2 Priority career advancement.

To test the career advancement mechanism, we created two dummy variables, *dtitle* and *dadministration*, corresponding to whether there was an increase in the doctor’s professional title and administrative level, respectively, to replace the dependent variable in the basic regression model. The results, as shown in columns (3) and (4) of Table 9, indicate that the treat×post coefficients for both *dtitle* and *dadministration* are significantly positive, with the coefficient for *dtitle* being larger. This suggests that doctors who volunteered in Wuhan are more likely to receive promotions in professional titles and administrative positions, thus increasing their reward.

### 5.7 Heterogeneity tests

#### 5.7.1 Gender effect.

Table 10 presents the estimation results for different genders, building upon the prior discussion of gender roles and their influence on job allocation and remuneration during public health emergencies. The results indicate that volunteering in Wuhan’s anti-epidemic rescue significantly enhances the future wages and performance for both male and female doctors. However, the increase in performance-based bonuses is significantly higher for male volunteers, confirming our expectations.

**Table 10:** The effect of gender.

	(1)	(2)	(3)	(4)	(5)	(6)
	*salary*		*bonus*		*salary_bonus*	
	Male	Female	Male	Female	Male	Female
*treat×post*	0.0855^**^	0.0767^***^	0.2290^***^	0.1306^**^	0.1916^***^	0.1358^***^
	(0.0364)	(0.0189)	(0.0606)	(0.0663)	(0.0419)	(0.0475)
*workload*	0.0045*	0.0038*	0.0494^***^	0.0573^***^	0.0393^***^	0.0419^***^
	(0.0023)	(0.0021)	(0.0072)	(0.0084)	(0.0051)	(0.0052)
*seniority*	-0.1068^***^	-0.0851^***^	0.4891^***^	0.4286^***^	0.3233^***^	0.2854^***^
	(0.0175)	(0.0193)	(0.0707)	(0.0716)	(0.0456)	(0.0442)
*age*	2.0291^***^	1.2902^***^	1.9996^**^	1.8232	1.6398^**^	1.3237*
	(0.3028)	(0.4604)	(1.0073)	(1.2550)	(0.6382)	(0.7326)
*_cons*	3.4201^***^	6.0449^***^	3.2255	4.0917	5.2435^**^	6.5236^**^
	(1.0846)	(1.6199)	(3.5936)	(4.4110)	(2.2784)	(2.5677)
Individual Fe	Yes	Yes	Yes	Yes	Yes	Yes
Year Fe	Yes	Yes	Yes	Yes	Yes	Yes
N	2014	1605	2014	1605	2014	1605
Adjusted ${\text{R}}^{2}$	0.9655	0.9546	0.7248	0.6515	0.8153	0.7586
_b[Male]-_b[Female]	0. 009		0. 098^**^		0. 056^**^	
P-value	0. 284		0. 026		0. 046	

#### 5.7.2 Educational background.

Based on whether doctors hold a Ph.D., we divided the sample into two groups: those without a Ph.D. (*Non*–*PhD*) and those with a Ph.D. (*PhD*). We anticipate that the impact of participating in the Wuhan anti-epidemic rescue efforts on career rewards will be more pronounced for doctors holding Ph.D. degrees. Table 11 presents the results for different educational levels, expanding on the notion that higher education equips doctors with superior skills and opportunities for advancement during public health crises. The results show that both Ph.D. and non-Ph.D. doctors experienced significant increases in their future payroll after participating in the Wuhan anti-epidemic rescue. However, the increase in performance-based bonuses was significantly higher for volunteer doctors with Ph.D. degrees compared to their peers without Ph.D. degrees, confirming our expectations.

**Table 11:** The effect of different educational background.

	(1)	(2)	(3)	(4)	(5)	(6)
	*salary*		*bonus*		*salary_bonus*	
	Non-Phd	Phd	Non-Phd	Phd	Non-Phd	Phd
*treat×post*	0.0830^***^	0.0604^**^	0.1627^***^	0.3104^***^	0.1466^***^	0.2619^***^
	(0.0262)	(0.0286)	(0.0505)	(0.0783)	(0.0338)	(0.0637)
*workload*	0.0046^***^	0.0023	0.0502^***^	0.0664^***^	0.0380^***^	0.0521^***^
	(0.0017)	(0.0037)	(0.0062)	(0.0103)	(0.0040)	(0.0074)
*seniority*	-0.1096^***^	-0.0834^***^	0.5291^***^	0.1701^**^	0.3549^***^	0.0918
	(0.0134)	(0.0263)	(0.0627)	(0.0810)	(0.0388)	(0.0563)
*age*	1.8239^***^	2.4694^***^	1.5893*	2.0800	1.2165^**^	2.1369^**^
	(0.2535)	(0.5728)	(0.8915)	(1.2544)	(0.5460)	(0.9408)
*_cons*	4.1733^***^	1.7881	4.6833	3.6263	6.7459^***^	3.9216
	(0.8973)	(2.0759)	(3.1371)	(4.5090)	(1.9207)	(3.3863)
Individual Fe	Yes	Yes	Yes	Yes	Yes	Yes
Year Fe	Yes	Yes	Yes	Yes	Yes	Yes
N	3028	591	3028	591	3028	591
Adjusted ${\text{R}}^{2}$	0.9608	0.9718	0.6783	0.8301	0.7826	0.8746
_b[Non-Phd]-_b[Phd]	0. 023^***^		-0. 148^***^		-0. 115^***^	
P-value	0. 000		0. 000		0. 000	

#### 5.7.3 Seniority effect.

We hypothesize that for doctors with more years of service, participation in the anti-epidemic rescue has a greater impact on salary returns. We divided the sample into two groups based on the median years of service: Low Seniority and High Seniority. Table 12 reports the estimation results for different levels of seniority. The results indicate that both lower and higher seniority doctors significantly improved their future wages and performance by participating in the Wuhan anti-epidemic rescue. However, the increase in performance-based bonuses was more pronounced for volunteers with higher seniority, confirming our expectations.

**Table 12:** The effect of different seniority.

	(1)	(2)	(3)	(4)	(5)	(6)
	*salary*		*bonus*		*salary_bonus*	
	Low	High	Low	High	Low	High
*treat×post*	0.0503^***^	0.0741*	0.1456*	0.2421^***^	0.1320^***^	0.2059^***^
	(0.0107)	(0.0399)	(0.0807)	(0.0412)	(0.0500)	(0.0367)
*workload*	0.0000	0.0103^***^	0.0564^***^	0.0449^***^	0.0420^***^	0.0347^***^
	(0.0007)	(0.0038)	(0.0069)	(0.0086)	(0.0046)	(0.0056)
*seniority*	-0.0181*	0.0279	0.3774^***^	-0.1407	0.2947^***^	-0.0775
	(0.0098)	(0.0867)	(0.0670)	(0.2233)	(0.0426)	(0.1608)
*age*	-1.4176^***^	3.0743^***^	3.2124	2.9410^***^	2.5969*	2.6960^***^
	(0.4237)	(0.3667)	(2.5879)	(0.8434)	(1.5237)	(0.6376)
*_cons*	15.3061^***^	-0.9595	-0.3904	1.1223	2.217	2.1882
	(1.4706)	(1.1902)	(8.9795)	(2.6913)	(5.2864)	(2.0530)
Individual Fe	Yes	Yes	Yes	Yes	Yes	Yes
Year Fe	Yes	Yes	Yes	Yes	Yes	Yes
N	1748	1716	1748	1716	1748	1716
Adjusted ${\text{R}}^{2}$	0.9683	0.9313	0.6239	0.8145	0.6976	0.8608
_b[Low]-_b[High]	-0.024		-0.097^**^		-0. 074^**^	
P-value	0. 16		0. 036		0. 022	

## 6 Conclusion and discussion

The institution of judicious reward mechanisms is imperative for safeguarding public health and societal stability, serving to invigorate healthcare professionals and refine public health emergency responsiveness through the assurance of frontline staff stability and contentment. Utilizing individual doctor data from a major hospital in China, spanning from 2017 to 2023, our empirical inquiry scrutinizes the impact of Wuhan anti-epidemic rescue participation on volunteer doctors’ remunerative outcomes, bolstering the comprehension of voluntary service and professional recompense interrelations.

Our study substantiates those doctors received commensurate rewards subsequent to their voluntary involvement in the Wuhan anti-epidemic rescue, manifesting as a marked salary increment relative to their non-participating peers. The persistence of this remuneration differential validates that the compensatory measures implemented by policymakers during the pandemic not only took effect but also achieved the intended positive impact. For the healthcare professionals, this represents a material acknowledgment of their dedication and sacrifices. Mechanism testing reveals that doctors who participated in the pandemic relief tended to be assigned to jobs requiring higher skills, reflecting an ascension in their professional capabilities. It also suggests that the increase in remuneration may be attributed to elevation in professional titles and administrative positions. Additionally, we find that specific demographics, such as male, highly educated, and senior healthcare professionals, experienced more pronounced increases in base salary and performance bonus, which may be correlate with their inherent advantages in professional competencies and responsibilities.

Our study transcends the examination of remuneration shifts post-volunteerism, embedding the phenomenon within the analytical ambit of health economics. It elucidates the mechanisms and outcomes of career “rewards” for volunteer doctors, offering actionable insights and theoretical scaffolding for policy design in forthcoming public health crises. First, policies should focus on supporting volunteer services, particularly by promoting crisis response education among medical students. Research indicates that medical students’ volunteer efforts during the pandemic are driven not only by altruism but also by their pursuit of professional identity and self-development [41]. Policymakers might consider introducing “Public Health Emergency Preparedness” courses into medical education to enhance students’ engagement and response capabilities in future public health crises.

Second, when formulating incentive policies for doctors, it is crucial to account for their intrinsic motivations, especially altruistic ones. Over-relying on extrinsic incentives, such as financial rewards, may undermine doctors’ intrinsic motivations, leading to decreased job satisfaction and professional loyalty [42]. Therefore, policies should strive to balance intrinsic and extrinsic motivations in their design. For instance, in addition to providing financial incentives, policies could enhance doctors’ sense of professional accomplishment and social recognition by acknowledging and honoring their contributions during crises. Future research should explore the differential impacts of various reward mechanisms, identifying which strategies most effectively foster both long-term intrinsic motivation and external performance. This will help in formulating more nuanced, effective policies that honor the contributions of healthcare professionals, particularly those combating pandemics.

While our data is sourced from a single hospital, this institution holds a prominent position in the local healthcare system, which makes its staff’s behavior somewhat representative. However, we acknowledge the generalizability limitations and call for further research in diverse contexts to validate and extend these findings. Future studies should examine similar phenomena in different healthcare settings and regions to determine whether these findings hold true in broader contexts and across varying health system structures. This will allow for greater validation and extension of our conclusions and provide a more comprehensive understanding of how voluntary participation in crises influences healthcare professionals’ careers.

## Supporting information

S1 File Additional tests. (PDF)

S2 File Research dataset. (XLSX)
