# Supplementary material for: From the frontlines to the future: Anti-epidemic volunteer experience and career rewards for doctors
Source: PLoS One. 2025 Jul 22;20(7):e0328044. doi: 10.1371/journal.pone.0328044 (PMC12282918; doi:10.1371/journal.pone.0328044)
Supplement: S1 File — (PDF) [file pone.0328044.s001.pdf]

Additional Tests

A1. PSM-DID

We compared the characteristics of the treated and control groups before the COVID-19 pandemic, as shown in **Table A1**. Compared to doctors without anti-epidemic volunteer experience, those with such experience differed in terms of salary, title, and education, but exhibited no significant differences in other characteristics. To better estimate the impact of anti-epidemic volunteer experience on doctors' remuneration, we employed the PSM-DID method to mitigate potential selection bias. We used one-to-one nearest neighbor matching with replacement, then tested the balance of control variables before and after matching.

Table A1. Comparison of Characteristics before the Shock

varname	obs(0)	mean(0)	obs(1)	mean(1)	mean-diff	t
salary	1323	10.4700	126	10.3568	0.1132***	3.2552
bonus	1323	11.8395	126	11.8362	0.0033	0.0505
salary_bonus	1323	12.1009	126	12.0718	0.0291	0.5814
workload	1323	5.6475	126	5.2576	0.3900	1.5691
seniority	1323	2.0686	126	1.9449	0.1237	1.4002
title	1323	3.1595	126	3.0000	0.1595*	1.8136
administration	1323	0.0612	126	0.0159	0.0454	1.4361
education	1323	1.7596	126	1.9603	-0.2007***	-3.0895
gender	1323	0.4346	126	0.4365	-0.0019	-0.0409

Table A2 presents the results of the balance tests, showing the differences between unmatched and matched samples. According to Rosenbaum and Rubin [24], the absolute value of the standardized bias should be less than 20% to achieve a satisfactory matching effect. Our results indicate that after matching, the absolute values of the standardized biases of the matched variables are all below 10%, meeting the matching requirements. Fig A1 shows the changes in standardized differences before and after matching. The significant reduction in these differences, mostly centered around zero, indicates good matching quality. Fig A1 shows that the propensity score distributions of the treated and control groups are more consistent after matching.

Table A2. Balance Test

Variable	Unmatched	Mean		%bias	%reduct	t-test		V(T)/
	Matched	Treated	Control		bias	t	p>t	V(C)
workload	U	5.5939	6.0069	-18.2		-1.12	0.261	0.88
	M	5.5939	5.4075	8.2	54.9	0.37	0.713	0.75
seniority	U	2.0752	2.1627	-11.3		-0.64	0.523	0.52*
	M	2.0752	2.0598	2	82.4	0.09	0.927	0.50*

title	U	3.0455	3.2338	-19.3		-1.14	0.255	0.7
	M	3.0455	3.0909	-4.6	75.9	-0.22	0.827	0.71
administration	U	.04545	.17532	-28.3		-1.48	0.141	0.27*
	M	.04545	.04545	0	100	0.00	1.000	1
gender	U	.43182	.43506	-0.7		-0.04	0.967	.
	M	.43182	.40909	4.6	-600	0.21	0.831	.

Note: "Mean" represents the means of the treated and control groups, "%bias" represents the standardized bias before and after matching, "%reduct |bias|" represents the percentage reduction in the absolute value of the standardized bias, and "t-test" indicates whether there are significant differences in the matched variables between the treated and control groups after matching.

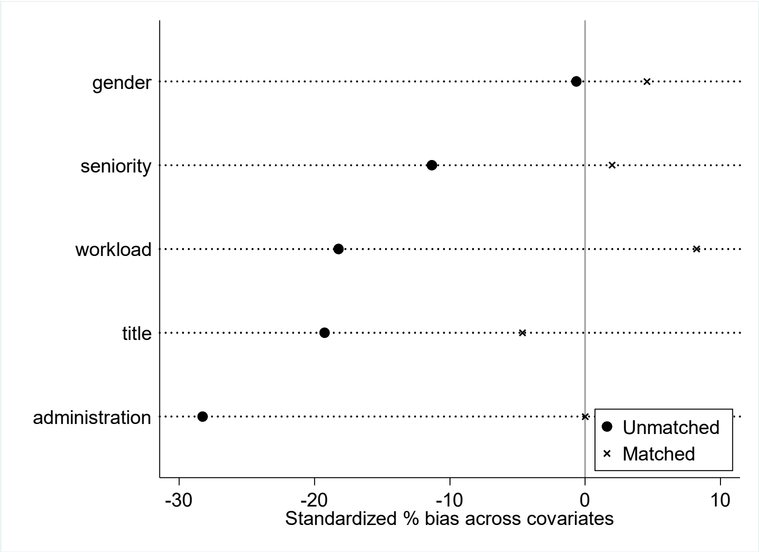

Fig A1. Standardized % Bias across covariates

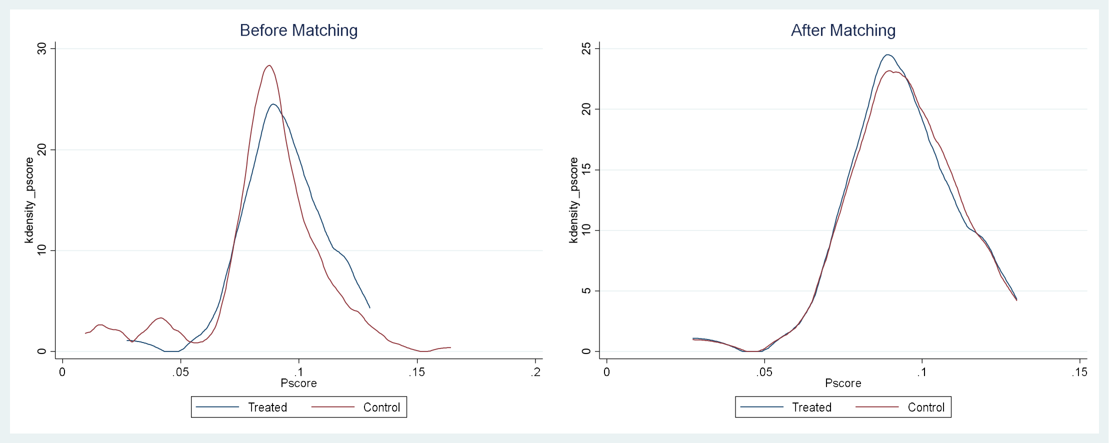

Fig A2. Kernel Density

Having satisfied the balance tests, we re-estimated the matched samples using the DID model, with results presented in **Table A3**. The coefficient of the interaction term of interest remains significantly positive, indicating that doctors with anti-epidemic volunteer experience receive more positive returns. Additionally, we employed nearest neighbor matching with replacement at ratios of 1:2 and 1:3 to obtain new samples and estimate the impact of anti-epidemic volunteer experience on doctors' salaries. The results were consistent with our expectations.

Table A3. PSM-DID Test

	(1)	(2)	(3)
	salary	*bonus*	*salary_bonus*
treat×post	0.0911***	0.1217	0.1340**
	(0.0261)	(0.0709)	(0.0483)
workload	0.0064	0.0279**	0.0239**
	(0.0056)	(0.0129)	(0.0103)
seniority	-0.1091***	0.4607	0.2689*
	(0.0259)	(0.2718)	(0.1506)
age	0.7532	5.5193	4.0530
	(0.9319)	(4.2843)	(2.3499)
_cons	7.9899**	-9.0211	-3.0925
	(3.3081)	(14.9046)	(8.2002)
Individual Fe	Yes	Yes	Yes
Year Fe	Yes	Yes	Yes
N	536	536	536
Adjusted R^2	0.9186	0.6550	0.7520

Table A4. PSM-DID Test with 1:2 Matching

	(1)	(2)	(3)
	salary	*bonus*	*salary_bonus*
treat×post	0.0857***	0.2045***	0.1774***
	(0.0247)	(0.0573)	(0.0383)
workload	0.0036	0.0458***	0.0356***
	(0.0039)	(0.0120)	(0.0095)
seniority	-0.0674**	0.6448***	0.4016***
	(0.0237)	(0.0856)	(0.0499)
age	0.5697	-0.7805	-0.0064
	(0.5659)	(1.3279)	(0.7562)
_cons	8.6034***	13.0722**	11.1108***
	(2.0126)	(4.7136)	(2.7168)
Individual Fe	Yes	Yes	Yes

Year Fe	Yes	Yes	Yes
N	812	812	812
Adjusted R^2	0.9482	0.6591	0.7610

Table A5. PSM-DID Test with 1:3 Matching

	(1)	(2)	(3)
	salary	*bonus*	*salary_bonus*
treat×post	0.0752***	0.2028***	0.1755***
	(0.0218)	(0.0533)	(0.0347)
workload	0.0056	0.0429***	0.0341***
	(0.0045)	(0.0115)	(0.0092)
seniority	-0.0858**	0.6364***	0.4033***
	(0.0338)	(0.0769)	(0.0456)
age	0.7735	-0.3564	0.0727
	(0.5828)	(1.3787)	(0.7833)
_cons	7.9120***	11.5395**	10.8045***
	(2.0742)	(4.9023)	(2.8053)
Individual Fe	Yes	Yes	Yes
Year Fe	Yes	Yes	Yes
N	996	996	996
Adjusted R^2	0.9326	0.6491	0.7522

A2. Placebo Test

The result, displayed in Fig 3, show that the coefficients for the interaction term treat×post are clustered around zero and markedly deviate from the actual regression coefficient, which is represented by the red vertical line. Most of these coefficients' p-values are above 0.1, confirming that the notable increase in wages for volunteer doctors is indeed a result of their participation in the response efforts and not due to random factors.

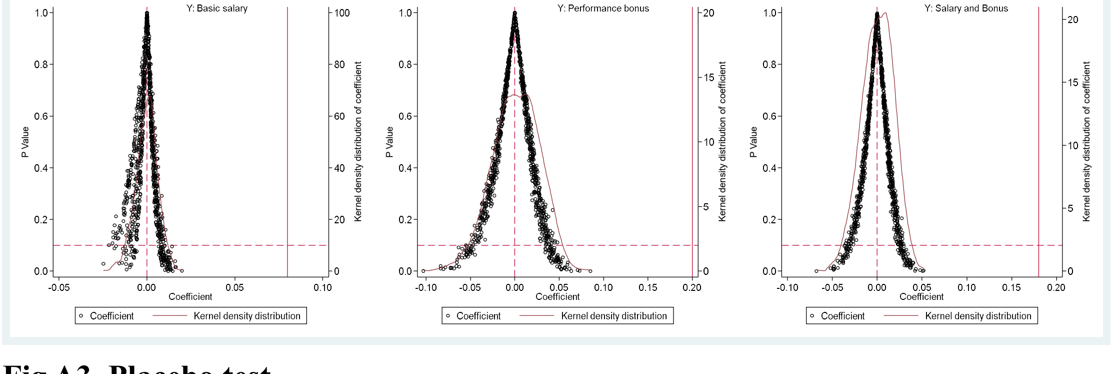

Fig A3. Placebo test
